# P-521. Assessing Infectious Disease Screening and Prevention Practices for Persons who Inject Drugs in a Large Midwestern Academic Medical Center

**DOI:** 10.1093/ofid/ofae631.720

**Published:** 2025-01-29

**Authors:** Rosemary C Bailey, Jessica S Tischendorf, Fauzia Hollnagel

**Affiliations:** UW Health - Madison, WI, Madison, Wisconsin; University of Wisconsin School of Medicine and Public Health, Madision, Wisconsin; UW Madison, Madison, Wisconsin

## Abstract

**Background:**

Persons who inject drugs (PWID) are at risk for infection, including HIV, hepatitis C virus (HCV), and sexually transmitted infections (STIs). Harm reduction counseling, HIV pre-exposure prophylaxis (PrEP) and post-exposure prophylaxis (PEP), and routine STI screening are recommended to prevent infectious complications among those with substance use disorder (SUD). Hospitalizations serve as an opportunity to provide these infection prevention and screening interventions which are often difficult to access in the ambulatory or community setting for patients with active SUD. We sought to describe the frequency of infectious disease screening and preventive counseling and interventions among PWID admitted to our hospital with a serious injection related infection (SIRI).

**Methods:**

We conducted a retrospective chart review of PWID > 18 years of age admitted to our large tertiary medical center in the Midwest with SIRIs in 2022. The conditions included were bacteremia, endocarditis, skin and soft tissue infection, epidural abscess, septic arthritis, osteomyelitis and other. We assessed the frequency of screening for HIV, hepatitis C, gonorrhea, chlamydia, and syphilis, and the frequency of harm reduction counseling and assessment for HIV-PrEP and PEP eligibility. We collected demographic information, discharge location and conditions, and whether infectious disease and addiction medicine were consulted. Descriptive analysis was performed.

**Results:**

45 patients with SIRIs were admitted in 2022. Of the 45, 56% underwent HIV screening, 53% HCV screening, and 16% received gonorrhea, chlamydia, and syphilis screening during the index hospitalization. Harm reduction counseling was documented for 69% of patients and was almost exclusively documented by addiction medicine. There was no documentation of PrEP consideration despite 36 patients meeting eligibility and no PrEP or PEP was prescribed.

**Conclusion:**

In our institution, there were suboptimal rates of infectious disease screening, harm reduction counseling, and HIV prevention interventions among PWID admitted with SIRI in 2022. The most marked gap in care is regarding consideration for HIV-PrEP. Further work will inform barriers and facilitators to optimizing these practices in the hospital setting.

**Disclosures:**

**Jessica S. Tischendorf, MD, MS**, Merck: Grant/Research Support

